# Exploring the Role of Determinants Affecting Responsible Underwater Behaviour of Marine-Based Tourists

**DOI:** 10.3390/bs14020141

**Published:** 2024-02-16

**Authors:** Ke Zhang, Lewis T. O. Cheung, Theresa W. L. Lam, Anson T. H. Ma, Lincoln Fok

**Affiliations:** 1 Department of Science and Environmental Studies, The Education University of Hong Kong, Hong Kong, China; kezhang@s.eduhk.hk; 2 York Business School, York St. John University, Lord Mayor’s Walk, York YO31 7EX, UK; 3 School of Agriculture, Food and Ecosystem Sciences, The University of Melbourne, Burnley Campus, 5500 Yarra Boulevard, Richmond, VIC 3121, Australia; theresa.lam@student.unimelb.edu.au; 4 Fenner School of Environment and Society, Australian National University, 141 Linnaeus Way, Canberra, ACT 2601, Australia; u7583673@anu.edu.au

**Keywords:** responsible underwater behaviour, diving attitude, diving experience, demographic features, recreational divers, Hong Kong

## Abstract

This study utilised divers’ demographic characteristics, diving experience, and attitudes to analyse the association between these factors and divers’ responsible underwater behaviour among Chinese scuba divers in Hong Kong. More innovatively, the measurement construct of diving attitude was further employed as a mediator to investigate its influence on the relationship between divers’ diving experience and responsible underwater behaviours based on the conceptual framework of previous works in the literature. The questionnaire data for this study were collected at four of the most popular dive sites among the marine protected areas in Hong Kong, with 398 valid samples after eliminating incomplete questionnaires. Regression results demonstrated that divers’ demographic characteristics could significantly predict their responsible underwater behaviour, with age (*b* = 0.10, *p* < 0.05) and education (*b* = 0.15, *p* < 0.05) being found to be positively associated with their diving behaviour. In addition, path analysis demonstrated that divers’ diving experience and attitude could explain 13.6% and 22.6% of the variance in predicting their responsible diving behaviour, respectively. However, no mediation effect was found on the relationship between diving experience and responsible underwater behaviour relative to diving attitude, given the absence of statistical effects regarding the positive impact of divers’ diving experience on their attitude (*β* = 0.024, *se* = 0.022, *t* = 1.085, *p* = 0.279). Based on the research findings, theoretical and practical implications were discussed correspondingly, which are believed to be beneficial in promoting marine conservation and the sustainable development of marine-based nature tourism in Hong Kong.

## 1. Introduction

Since the 1970s, scuba diving has transformed remarkably, emerging from a relatively niche recreation to a prominently visible and widely accessible water-based activity across the globe [1]. However, the increased accessibility and popularity of scuba diving have caused significant coral reef damage despite its considerable financial success throughout the recreation process [2,3].

Given the negative aspects of scuba diving in terms of marine conservation, studies have recommended various management frameworks to monitor and mitigate the potentially detrimental consequences of scuba diving on marine ecological sustainability, such as by facilitating the environmentally responsible diving programme, predive briefing, visitation monitoring programs, an educational video briefing program, and direct underwater reinforcement [2,4,5,6,7,8,9,10]. Despite the effectiveness of these measurements being commonly acknowledged in dealing with the preservation of marine ecology, scholars have revealed that divers play a crucial role in contributing to marine conservation. Particularly, Hammerton and Bucher [6] revealed that the extent to which marine ecosystems are impacted depends on divers’ responsible underwater behaviour, as divers’ contact or their used diving equipment is a primary mechanism for chronic impact on benthic life forms. Under these circumstances, divers’ responsible underwater behaviours have been constantly investigated through a variety of research attributes, including diving experience [1,2,4,11,12,13], diving attitude [12,14,15,16,17], sociodemographic characteristics [8,11,18,19], and their levels of specialisation [20,21,22] across various diving locations.

For instance, Hammerton [1] pointed out that divers’ responsible diving behaviours could significantly vary based on their experience levels. Australian divers who had abstained from diving for 6 to 12 months exhibited less responsible behaviour (the frequency of diver contacts with reef biota) than divers who had engaged in diving within the previous 24 h. Likewise, studies have also shown that divers’ responsible diving behaviours might differ significantly according to their sociodemographic status. In particular, Mcbride [19] and Lucrezi, Ferretti, Milanese, Sarà and Palma [17] found that younger divers perceive more responsible diving behaviour than older individuals. At the same time, Chung, Au and Qiu [18] and Worachananant, Carter, Hockings and Reopanichkul [4] indicated that male divers are more responsible for their underwater diving behaviour than female divers.

In addition to the diving experience and demographic variables, Ong and Musa [12] concluded that divers’ diving attitude could predict their responsible diving behaviour and partially mediate the relationship between divers’ diving experience and their responsible underwater behaviour. This suggests that divers with a higher level of diving experience may have a better attitude and, hence, achieve a higher level of responsible diving behaviour. However, although the information accumulated from these previous investigations is informative, there remains a lack of understanding regarding the specific factors that affect divers’ responsible diving behaviour in Hong Kong. In particular, a study conducted by Chung, Au and Qiu [18] using divers’ diving experience and demographic attributes to understand divers’ diving behaviour in Hong Kong was based on the direct observation method with a sample size of just 81 recreational divers. Likewise, the latest study by the World Wide Fund for Nature (WWF) examined the responsible diving behaviour of local divers in Hong Kong, which is more focused on direct observation through a citizen science approach to collect the research data [23]. In addition to the difference in methodology design, little has been reported in examining a structured relationship between divers’ diving experience and their responsible diving behaviour utilising diving attitude as a mediator in studying Chinese scuba divers in Hong Kong. Acknowledging the existing research gap in the literature, especially concerning the absence of assessing the relationship between divers’ diving experiences and their responsible diving behaviour by introducing diving attitude as a mediator variable among Chinese scuba divers in Hong Kong, this study aims to address such a gap by developing a structural equation model to explore the possible effects of divers’ diving attitude in mediating the relationship between divers’ diving experiences and their responsible underwater activities. By performing the statistical analysis, the present study seeks to contribute significant insights to fill the current research gap in the literature, particularly helping to strengthen stakeholders’ comprehension of potential factors in shaping divers’ responsible diving behaviour in the event of supporting the development of marine ecology conservation and scuba diving tourism sustainability in Hong Kong.

Therefore, by taking into consideration the importance of divers’ demographic characteristics, diving experience, and diving attitude contributing to the impact of their responsible underwater diving behaviour, as well as the perceived theoretical research gaps in the literature, four research questions were proposed to investigate the potential effects of divers’ sociodemographic attributes, diving experience, and diving attitude on their responsible underwater behaviour among Chinese scuba divers in Hong Kong.

- Q1: Do divers’ demographic characteristics correlate positively with their responsible underwater behaviour while diving in Hong Kong?
- Q2: Are divers’ diving experiences positively or negatively associated with their responsible diving behaviour in Hong Kong?
- Q3: What is the relationship between divers’ diving attitude and their responsible diving behaviour in Hong Kong?
- Q4: Is the relationship between divers’ diving experience and their responsible diving behaviour mediated by the level of divers’ diving attitude while diving in Hong Kong?

Based on how the research questions proposed, the present study is expected to achieve both practical and theoretical contributions to support sustainable scuba diving tourism and marine-based ecological conservation in Hong Kong by achieving four research objectives: (1) investigating the relationship between divers’ sociodemographic characteristics and their responsible underwater behaviour; (2) assessing the relationship between divers’ diving experience and their responsible underwater behaviour; (3) analysing the relationship between divers’ diving attitude and their responsible underwater behaviour; and (4) exploring the mediating role of diving attitude in the relationship between diving experience and divers’ responsible underwater behaviour. Practically, this study’s findings are expected to provide recommendations related to management implications for assisting stakeholders in establishing effective policies and practices to promote the sustainable development of scuba diving tourism and marine-based ecological preservation, especially concerning the management implications for those ecologically sensitive marine species, such as coral reefs. This is because sustainable scuba diving tourism is inevitably linked to or inseparable from integrating comprehensive insights into divers’ responsible underwater behaviour concerning ecological preservation, as favourable marine ecology conditions free damage from divers’ underwater diving behaviour could contribute significantly to enhancing divers’ satisfaction to attract divers’ continued motivation and confidence to participate in scuba diving recreation, thus promoting further financial sustainability within the tourism industry [6,24]. In addition to the practical contribution, the results of the present study are expected to confirm further measurement factors that influence divers’ responsible underwater behaviour to enrich the theoretical knowledge of nature-based tourism while helping to fill the research gaps in the current literature regarding the sustainable development of marine-based nature tourism in China.

## 2. Conceptual Framework

### Model Specification

**Figure 1:**
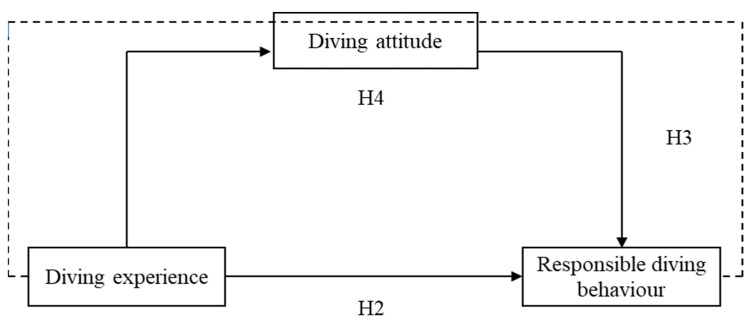
Conceptual framework.

The research framework of this study was developed based on a comprehensive review of the previous scuba diving literature (Figure 1). The framework consisted of three independent variables, namely, divers’ demographic characteristics, diving experience, and diving attitudes, while the variable of responsible underwater behaviour was proposed as the dependent variable. Regarding the association between divers’ demographic features and their responsible underwater behaviour, Giglio, Marconi, Pereira-Filho, Leite, Figueroa and Motta [8] and Mcbride [19] discovered an apparent correlation between divers’ gender and age variations throughout their responsible underwater diving behaviour in Brazil and Seychelles. They found that females and younger divers generally demonstrated more responsible diving behaviour while diving. Thus, it is likely that there might be a direct relationship between demographic attributes and responsible underwater behaviour among scuba divers in Hong Kong. In addition to the perceived correlations between divers’ demographic features and their responsible diving behaviour, a direct association between divers’ diving experience and their responsible underwater behaviour was observed based on the findings of Ahmad Puad and Khairul Amri [13], who indicated that increasing diving experience (increasing number of logged dives) would result in a gradual reduction of divers’ damaging underwater contact behaviour. Hence, it is reasonable to hypothesise that there might be a direct relationship between divers’ diving experience and their responsible diving behaviour among scuba divers in Hong Kong. Interestingly, in understanding factors influencing Malaysian divers’ underwater responsible behaviour, Ong and Musa [12] proposed that divers’ diving experience could be indirectly associated with their responsible diving behaviour through their diving attitude. This means that diving attitude could mediate the relationship between diving experience and responsible underwater behaviour. In addition to understanding the effect of employing the mediator of diving attitude on assessing the relationship between divers’ diving experience and their responsible diving behaviour, Ong and Musa [15] further extended their explorations by integrating the mediator of diving attitude to discover the relationship between divers’ environmental concern and their responsible underwater behaviour. They found that a partial mediation between divers’ environmental concern and their underwater behaviours was confirmed for the model, indicating that both diving attitude and environmental concern could explain the changes in the variance of scuba divers’ responsible diving behaviour while diving in Malaysia. These discoveries have significantly underscored the crucial role of divers’ diving attitudes in comprehending and mediating the relationship between potential factors associated with divers’ recreational engagements and their responsible diving behaviour. Logically, these results might have also significantly underscored the importance of an individual’s attitude as a critical factor in influencing tourists’ behaviours across different research domains in the tourism development process, including, but not limited to, the research context of scuba diving. Against this background, scholarly investigations through different research models and conceptual frameworks have been constantly explored, and it has been revealed that enhanced tourists’ recreational attitude might encourage recreationists to take concrete action for ecological conservation and support for pro-tourism behaviour [22,25,26,27]. For instance, Erul, Uslu, Cinar and Woosnam [26] proposed a value–attitude–behaviour (VAB) model to understand Manavgat residents’ supporting behaviour for tourism development in Turkey during the COVID-19 pandemic. They found that residents’ welcoming nature could strongly indicate their support and participation in tourism-related activities during the pandemic, further emphasising the significance of individuals’ attitudes in shaping human behavioural intention in the tourism literature. Nevertheless, despite the growing body of research on investigating the association between tourist attitude and perceived behavioural intention, there is still a noticeable absence of empirical studies in the tourism literature regarding the mediating impact of attitude towards tourist behaviour [27]. To remedy this shortcoming, Hasan, Ray and Neela [27] proposed the mediator of tourist attitude to test the relationship between destination imaging, perceived value, perceived risks, and satisfaction towards their behavioural intention to visit the coastal travel destinations in Bangladesh, and they found that tourists’ attitudes could mediate the relationship between destination image, perceived value, satisfaction, and behavioural intention to visit coastal tourism destinations. Significantly, the research findings of Hasan, Ray and Neela [27] have helped to fill a theoretical gap in understanding tourists’ behavioural intentions by utilizing the mediator of attitude in the context of beach tourism, which is believed to be able to enrich and expand new conceptual perspectives to understand tourist attitude and behavioural intention relation in attitude–behaviour theories across various research domains within outdoor recreation tourism, including, but not limited to, the coastal tourism settings. Under these circumstances, the present study hypothesizes that diving attitude could act as a mediator in understanding the relationships between diving experience and responsible underwater behaviour among recreational divers in Hong Kong. The following four hypotheses are constructed within the conceptual framework based on the aforementioned scientific evidence.

**Hypothesis** **1.** *There is a positive relationship between divers’ demographic characteristics and their responsible underwater behaviour.*

**Hypothesis** **2.** *A positive relationship exists between divers’ diving experience and their responsible underwater behaviour.*

**Hypothesis** **3.** *A positive relationship is present between divers’ diving attitude and their responsible underwater behaviour.*

**Hypothesis** **4.** *Diving attitude mediates the relationship between divers’ diving experience and their responsible underwater behaviour.*

## 3. Methodology

### 3.1. Study Areas

With the successful establishment of the first batch of marine parks in July 1996, seven marine parks and one marine reserve were developed in Hong Kong. Marine parks serve an essential role as protected areas that provide conservation, education, and recreation opportunities for the general public to appreciate and support marine conservation [28,29,30]. Among these functions of designated marine parks, the incredible recreation opportunities offered by marine parks with unrestricted tourist visitation have allowed many recreationists to undertake water-based activities, such as diving, canoeing, swimming, and snorkelling. Scuba diving is one of the most popular water-based activities that has emerged in marine protected areas in Hong Kong, where the eastern and northeastern parts of marine waters aggregate most recreational divers, with approximately 33 diving sites [18]. Divers choose to dive in the eastern waters of Hong Kong because of the prosperous coral communities along with its better underwater visibility compared with other waters in Hong Kong [24]. Thus, four of the diving hotspots across eastern and northeastern waters in Hong Kong were selected as the questionnaire survey sites, including Tung Ping Chau, Hoi Ha Wan, Sharp Island, and the Ung Kong group. These sites serve as the most popular diving locations, attracting the most divers and allowing a better representation of the general scuba diver population across Hong Kong.

### 3.2. Development of the Research Instrument

This study used a structured questionnaire as the primary data collection method to explore and answer the research questions. The questionnaire consisted of four sections that measured the variables of divers’ demographic features, diving experience, diving attitudes, and responsible diving behaviour. Measurement items in the questionnaire were developed based on a comprehensive review of the relevant literature with slight modifications to comply with the local situation for the target population in Hong Kong. The first section contains several demographic questions regarding information on the gender, age, education level, and monthly income of the individual respondent. The second section contained four measuring items representing the construct of divers’ experience level, which was defined based on the methodology of several previous studies [11,13,24,31]. These items characterized the diving experience according to divers’ level of involvement, frequency of participation, and levels of diving certification, aiming to evaluate a diver’s past diving engagements comprehensively. The third section had three items in the explanation for the measurement construct. It was taken and modified from a previous study by Luo, et al. [32] to measure divers’ attitudes toward scuba diving recreation. To ensure the effectiveness and accuracy of the measurement, the present study interviewed some experienced divers while referring to the general management guidelines before designing and confirming the measurement items for diving attitudes. Not surprisingly, both divers and the management guidelines indicated that a good diving attitude goes along with a good sense of public responsibility for protecting the marine environment while diving in marine areas. Under these conditions, the three measurement elements of diving attitude were developed based on divers’ self-opinions and the code of conduct to evaluate their public responsibility for preserving the marine environment while diving in Hong Kong. The final section measured the construct of responsible underwater behaviour with four items to understand divers’ diving behaviour concerning marine conservation, which was adapted from the previous literature [16,20]. These four behavioural items are designed with a slight modification to focus on self-reported behaviour concerning the responsible behaviour of divers. 

### 3.3. Sampling and Data Collection

**Figure 2:**
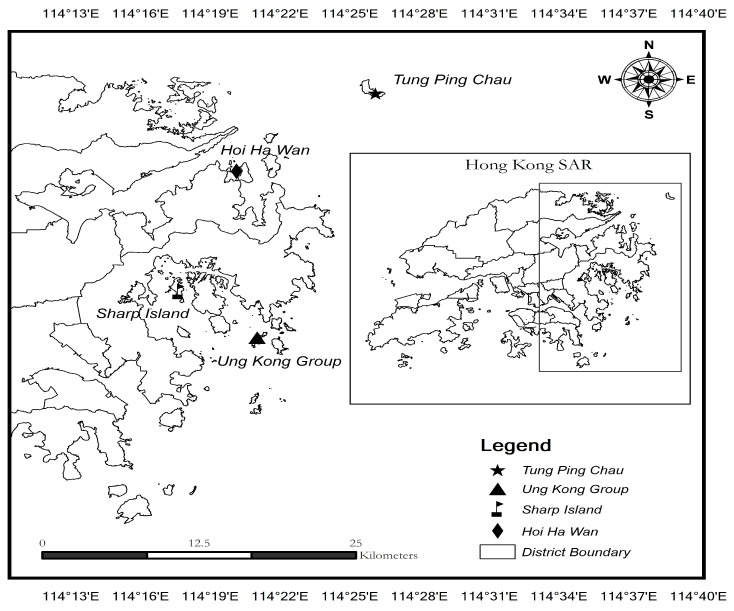
Sites of questionnaire surveys.

The population under investigation was the scuba divers diving the popular diving hotspots in the eastern and northeastern waters of Hong Kong, including Hoi Ha Wan Marine Park, Tung Ping Chau Marine Park, Sharp Island, and Ung Kong Group (Figure 2). The data were collected between the summers of 2021 and 2022 using a convenience sampling approach to survey the target recreational divers around the selected diving sites. Prior to the official commencement of the data collection procedure, a pilot study was randomly conducted with a small group of recreational divers to help this study identify possible failures and inappropriate measurement designs before implementing the full-scale investigation [33]. Specifically, the selected divers were invited to respond to the drafted questionnaires and request feedback regarding assessing the clarity and relevance of the indicators based on their experiences and perceptions after answering the questionnaires. At the same time, reviews and suggestions from academic experts and stakeholders involved in the scuba diving industry were theoretically and practically evaluated to ensure the validity and appropriateness of the proposed measurement constructs for the questionnaires were under the right track. These comprehensive evaluation processes were undertaken before finalizing the research questionnaires, aiming to enhance the overall reliability of the measurement instrument for the present study. During the data collection phase, a convenience sampling method was adopted for the simplicity of its operating practices and the advantages of saving time and expenses [34], considering that there may be some temporary changes in the data collection schedule and transportation arrangements concerning the weather and pandemic conditions. In addition, considering the difficulty of collecting questionnaires during the pandemic, the present study has sought assistance from various diving clubs and societies in Hong Kong to locate the target respondents and help from the personnel of different protected areas to facilitate the data collection process more flexibly.

The total sample size for this study was approximately 400, and the sample of these 400 scuba divers would yield a confidence level of 95% with a margin of error of 5% in the statistical calculation for achieving our study’s minimum required sample size [35]. Consequently, 550 questionnaires were printed for distribution in the selected diving sites, of which 398 were completed and returned, yielding a response rate of 76% after censoring and eliminating incomplete or invalid questionnaires.

### 3.4. Data Analysis

Based on the proposed research questions of this study, SPSS 28 was used to conduct descriptive statistical analysis, reliability, correlation, and exploratory factor analysis. In contrast, Amos 28 was used to perform the confirmatory factor analysis by verifying the proposed model’s factor structure and goodness of fit. After satisfying the predetermined statistical criteria of the proposed model in the CFA, multiple regression and a regression-based PROCESS method were used to analyse the hypothesis concerning the relationship between the independent and dependent variables and to assess the significance of the mediation relationship in this study.

## 4. Results

### 4.1. Descriptive Statistics

Table 1 summarizes the demographic features of this study’s respondents. Regarding the gender distribution of recreational divers, females comprise 51.5% of the sample, and males comprise 48.5%. More than 75% of respondents were between 18 and 39 years of age, with the 30–39 age group accounting for more than 30.9% of all responses. In terms of education level, more than 80% of the respondents had a bachelor’s degree or above, and approximately half had a monthly salary of 20,000 HKD or higher. By comparison, the remaining 13% of respondents were either retired or did not wish to disclose their economic status for the investigation.

### 4.2. Exploratory Factor Analysis

With the proposed methodology in mind, exploratory factor analysis was used to find common factors that explain the structure of the observed variables [36]. Traditionally, the Kaiser–Meyer–Olkin (KMO) test and Bartlett’s test of sphericity are the most common methods that help to confirm the suitability of using factor analysis and have been widely adopted by scholars across different research disciplines [36,37,38].

According to Choudhry, Fang and Lingard [36] and Kaiser [37], the acceptable interval for the Kaiser–Meyer–Olkin (KMO) values should be greater than 0.6, while the significance cutoff value for passing Bartlett’s test of sphericity should be less than 0.05. In this case, the KMO value for our study was 0.79, which is significantly higher than the suggested benchmark value of 0.60, as recommended by Kaiser [37] and Choudhry, Fang and Lingard [36], demonstrating that a sufficient number of measurement items for each component were presented in this study. In addition to the KMO value, Bartlett’s test of sphericity yielded a chi-square value of 1686.67 with 55 degrees of freedom (*p*-value = 0.000), indicating that the data do not generate an identity matrix or substantially deviate from identity [36]. Thus, these results could conclude that this study is appropriate for exploratory factor analysis, following its satisfying results of achieving the criteria of the KMO and Bartlett’s sphericity test.

In terms of how factor analysis works, this study used principal component analysis to group similar measurement items, followed by the variance rotation method to identify the factors with the appropriate measurement constructs to make the final principal components easier to understand [38]. Specifically, Olawale and Garwe [38] and Choudhry, Fang and Lingard [36] found that the number of possible latent factors could be determined using the number of measurement factors with an eigenvalue of one or more in the EFA. As such, three components or factors achieved an eigenvalue of one or more and were extracted from the 11 items in the questionnaires, including diving experience, diving attitude, and responsible underwater behaviour. Among these components, a four-item structural measurement confirmed that diving experience was the first factor to generate the highest explained variance (28.02%). Following this was a three-item structural measurement to prove that diving attitude was the second factor explaining (17.92%) of the total variance. Comparatively, a four-item structural measurement identified responsible diving behaviour as the last factor with the lowest explained variance (17.73%). Based on the confirmed measurement structures established by EFA, the internal consistency test was conducted for the three factors extracted in the exploratory factor analysis. The consistency coefficients of the three-factor construct were found to be greater than 0.6 to be above the recommended benchmark value suggested by Pallant [39] and Bagozzi and Yi [40], indicating that the proposed factor constructs were suitable for further statistical analysis (Table 2).

### 4.3. Measurement Model

Confirmatory factor analysis generates and assesses the measuring model based on the exploratory factor analysis’s three-dimensional factors. The construct factors were diving experience, attitude, and responsible diving behaviour, comprising 11 measurement items. These items were placed in separate measurement constructs and tested using the maximum likelihood (ML) estimate method to determine how well the measurement model fits the observed data [41].

Regarding the fit indices of measuring the proposed constructs, the CFA indicated that the *x*^2^ of the measurement model was 130.259, with 41 degrees of freedom (*df*) (*p* < 0.05); hence, the *x*^2^/*df* value fell within the range of 2 to 5, demonstrating that the measurement model provided a satisfactory fit to the data [42]. In addition to the chi-square statistics, alternative fit indices achieved a comparative fit index value of 0.946, a goodness-of-fit index of 0.945, a normed fit index of 0.924, and a root mean square error of approximation value of 0.074. These values are well above the benchmark thresholds suggested by scholars [43,44]. Thus, these findings demonstrate that the suggested three-factor model performed effectively with our study data.

### 4.4. Construct Validity

The final measurement model developed based on confirmatory factor analysis generally requires a construct validity assessment to determine whether the model can accurately represent and measure what is needed to conduct the investigation [45]. Correspondingly, several statistical conditions are commonly considered and applied to assess the construct validity of the study model before testing the research hypothesis, including unidimensionality, reliability, convergent validity, and discriminant validity [45].

Regarding the results of unidimensionality (Table 3), the regression weights achieved factor loadings for the 11 items ranging from 0.45 to 0.95, which is greater than 0.4 according to the suggested value of Hulland [44], indicating that the unidimensionality was established in this study. In addition, the composite reliability for all the construct variables was above the recommended criterion of 0.6, with values ranging from 0.64 (responsible underwater behaviour) to 0.90 (diving experience) to surpass the recommended cutting thresholds based on the findings of Bagozzi and Yi [40]. This demonstrates that the measurement constructs were reliable and had tremendous internal consistency in the model. Similarly, the average variance extracted (AVE) value also demonstrated a significant result with the constructed variable of diving experience (0.69) and diving attitude (0.50) exceeding the recommended threshold of 0.5, as suggested by Fornell and Larcker [46] and Bagozzi and Yi [40] for assessing the proposed measurement constructs’ convergent validity. Despite these significances, the constructed measurement of responsible underwater behaviour (0.32) was deemed unsatisfactory because the AVE value was below 0.5. To remedy this restriction, Fornell and Larcker [46] indicated that the convergent validity of the measurement construct is still adequate if the composite reliability is greater than 0.6, even though the AVE value is less than 0.5, implying sufficient commonality between the different items in the measurement tool was established in our study. By the end of the validity assessment, the discriminant validity was achieved by comparing the square root of AVE values to the correlations between the target construct and the rest of the constructs, with a good level of validity reached when the square root of the AVE value of a particular construct variable is greater than the cross-correlation coefficient between that construct variable and other measurement constructs [46]. Table 4 shows that the square roots of the AVE for a given construct variable were all higher than their cross-correlation values with other constructs, indicating that discriminant validity was shown and established in the model [46]. However, despite the dominant effectiveness of the Fornell–Larcker criterion and the examination of cross-loadings perceived to the assessment of the discriminant validity, these methods are not consistently effective in identifying the absence of discriminant validity in typical research scenarios, particularly when identifying concerns with discriminant validity between latent measurement constructs [47]. To remedy this issue, Henseler, Ringle and Sarstedt [47] introduced a new methodology to the assessment of the discriminant validity known as the heterotrait–monotrait (HTMT) ratio of correlation, aiming to enhance a higher specificity and sensitivity in detecting the discriminant validity over the conventional methods of cross-loading criterion and Fornell–Lacker. Under this circumstance, the current study has decided to conduct additional examination by integrating the HTMT ratio of correlation alongside the assessment of the Fornell–Larcker criterion and the examination of cross-loadings to the assessment of the discriminant validity to make sure the latent measurement constructs of this study have a more refined and accurately designed conceptual framework before performing further statistical analysis. Correspondingly, the results of the HTMT analysis are presented in Table 5. The findings suggest that the proposed latent constructs of the current study had no discriminant validity concerns, as the threshold value of the HTMT for the three variables were all found to be below a strict criterion value of 0.850 and a liberal threshold value of 0.900 based on the assessment recommendation of Henseler, Ringle and Sarstedt [47]. In summary, the assessment of construct validity has demonstrated satisfactory reliability, convergent validity, unidimensionality, and discriminant validity, suggesting that the proposed construct variables are suitable for future investigations to address the research questions and help achieve our research objectives.

### 4.5. Hypothesis Testing

Multiple linear regression was employed to assess the first hypothesis to investigate the relationship between divers’ demographic features and their responsible underwater behaviour (Table 6). The results showed that divers’ demographic features could significantly predict their responsible underwater behaviour (*F* (4,393) = 3.79, *p* < 0.001); the four predictors together could explain 4% of the total variance in responsible underwater behaviour. In particular, age (*b* = 0.10, *t* = 2.55, *p* < 0.05, *β* = 0.14) and education (*b* = 0.15, *t* = 2.71, *p* < 0.05, *β* = 0.14) were positively associated with divers’ responsible underwater behaviour, whereas gender (*b* = 0.09, *t* = 1.24, *p* = 0.22, *β* = 0.06) and salary (*b* = −0.03, *t* = −1.92, *p* = 0.06, *β* = −0.10) were not associated with divers’ responsible underwater behaviour, suggesting that *H1* was principally supported. In addition to the demographic variables, Table 6 shows that divers’ diving experience could significantly and positively affect their responsible underwater behaviour (*β* = 0.228, *se* = 0.029, *t* = 7.903, *p* < 0.001), thus suggesting that *H2* was supported. Similarly, divers’ diving attitude is a positive predictor of their responsible underwater behaviour (*β* = 0.429, *se* = 0.063, *t* = 6.776, *p* < 0.001), thus demonstrating that *H*3 was also supported. By studying the last hypothesis concerning the mediation analysis, the proposed assessment for the mediation effects was similar to the method of Lombardi, et al. [48] by using the analytical framework of Baron and Kenny [49] and the PROCESS macro plugin of Hayes [50] to determine how the mediator of diving attitude affects the relationship between divers’ dive experience and their responsible underwater behaviour. These two different but complementary procedures were achieved based on the regression-based bootstrapping approach, which is highly specialized for mediation analysis [48].

With greater detail concerning the conditions for establishing a mediator, Baron and Kenny [49] indicated that the mediating effects could only be determined when the proposed model satisfied all three statistical conditions, including that the independent variable (*X*) must be a significant predictor of the dependent variable (*Y*), the independent variable (*X*) significantly affects the mediator variable (*M*), and the mediator variable (*M*) is a significant predictor of the dependent variable (*Y*), even after controlling for the effect of the independent variable (*X*). In comparison, Hayes [50] claimed that the PROCESS mediation effect shall be presented if the 95% confidence interval between the values of LLCI and ULCI does not contain zero. In contrast, an indirect effect is absent if the 95% confidence interval comprises zero for the interval value between LLCI and ULCI. According to Table 7, no mediating effect is found in this study because the regression results do not meet the second step of the mediation analytical framework, as suggested by Baron and Kenny [49]. This finding was enhanced by the PROCESS macro plugin of Hayes [50] because the 95% confidence intervals [LLCI, ULCI] = [−0.007, 0.028] for the indirect effect contain zero (Table 8), indicating that divers’ diving attitude could not function as a mediator in interpreting the relationship between divers’ diving experience and responsible underwater behaviour.

## 5. Discussion

This study developed four research hypotheses to understand how divers’ demographic features, diving experience, and attitudes influence their responsible underwater behaviour. It also investigated the relationship between diving experience and responsible underwater behaviour by introducing diving attitude as a mediator variable. In line with the first hypothesis, the present study found that divers’ responsible underwater behaviour differed significantly depending on their demographic features. Divers’ age and educational background were positive predictors of their responsible underwater behaviour, with older and well-educated divers behaving more responsibly than younger and less educated recreational divers diving in Hong Kong. These results were consistent with the previous findings of Musa, Seng, Thirumoorthi and Abessi [11] and Chung, Au and Qiu [18], who suggested that divers with a higher level of education and those under older age groups demonstrated less irresponsible diving behaviour. This could be because higher-income divers tend to be well-educated and may understand the importance of protecting the environment for the long-term sustainability of ecology and tourism development. As a result, they may behave more responsibly to avoid damaging the marine environment while diving. Along the same logic, older divers may be less likely than younger divers to participate in high-intensity water-based activities due to their busy work schedules or physical conditions. In this case, the older group of divers may record less irresponsible underwater behaviour than younger groups due to the lower frequency of participation in marine protected areas. However, the present study contradicted the findings of Mcbride [19] and Giglio, Marconi, Pereira-Filho, Leite, Figueroa and Motta [8], who indicated that significant differences between divers’ responsible diving behaviour and gender difference are perceived, with female divers demonstrated as more responsible than males while diving. The result discrepancy between the previous and present studies may be due to the differences in the sampling distribution. In particular, the studies of Giglio, Marconi, Pereira-Filho, Leite, Figueroa and Motta [8] and Mcbride [19] featured predominantly male divers, while the sample size for both males and females in the current research was evenly distributed.

Regarding the second hypothesis, this study’s result indicated that divers’ diving experience could significantly predict their responsible underwater behaviour, suggesting that divers with a higher level of diving experience were more environmentally responsible while diving. This finding was consistent with the results of Ong and Musa [12] and Lucrezi, Ferretti, Milanese, Sarà and Palma [17], who found that divers’ diving experience could influence their responsible underwater behaviour, with more experienced divers showing more responsible diving behaviour than less experienced divers. This could be because experienced divers may have more opportunities and abilities to see the wide variety of marine life to help them understand its vulnerability, making them behave more responsibly when diving in protected areas. Additionally, divers with increased diving experience generally acquire better diving skills (e.g., buoyancy and swimming technique), and these skills may support them in avoiding damaging marine ecosystems either intentionally or unintentionally. However, our study challenged the results of Camp and Fraser [5] and Hammerton [1], who found that experienced recreational divers appeared to behave no less irresponsibly than less experienced divers while diving. The discrepancy between the results of this study and previous studies may be related to the advancement of diving equipment, where the updating and improvement of diving equipment may directly or indirectly reduce irresponsible underwater behaviour by recreational divers on a technical level. In addition to the diving equipment upgrades, using additional equipment, such as photographic equipment, may lead experienced divers to behave less responsibly than those less experienced divers who did not equip photographic equipment due to the possible operational difficulties of holding the extra equipment while diving. This assumption is somewhat in line with the conclusion of previous findings indicating that photographers demonstrated less responsible underwater behaviour compared with those divers without using cameras while diving [1,17,19]. In terms of the third hypothesis, the current study demonstrated that divers’ diving attitude significantly predicted their responsible underwater behaviour, suggesting that divers with greater environmental awareness were associated with behaving more responsibly. This result was supported by Lucrezi, Ferretti, Milanese, Sarà and Palma [17] and Allkins, Tshipala and Hermann [16], who indicated that divers’ diving attitudes were a significant predictor of their responsible underwater behaviour in Italy and South Africa. This phenomenon may be perceived because divers with positive attitudes toward diving may also have more knowledge, commitment, and awareness of marine conservation, which helps them exhibit better and more skilful diving behaviours to avoid damaging marine biology [14]. However, the present study’s findings were in contrast to the observation of Lucrezi, Ferretti, Milanese, Sarà and Palma [17], who discovered that divers who claimed to keep a reasonable distance from bottom habitats made more involuntary contacts. The authors then explained that this result may have occurred because some divers overestimated their self-reporting skills, and their underwater responsible behaviour may not be ensured without solid diving skills despite the great diving intentions that could translate into responsible underwater behaviour. Significantly, these findings demonstrate that diving attitude is a complicated phenomenon that requires being determined based on various research contexts for management measures to be successfully handled and enforced.

Finally, in line with hypothesis four, this study’s findings did not support the hypothesis regarding the effectiveness of the proposed mediating variable of diving attitude in assessing the relationship between diving experience and responsible underwater behaviour. The finding of the present study has challenged the result of Ong and Musa [12] and Ong and Musa [15], who revealed that divers’ diving attitude could serve as a mediator in piratically mediating the level of relationship between divers’ diving experience and their responsible underwater behaviour, as well as divers’ environmental concerns and their responsible underwater behaviour. The discrepancy between the present and previous studies may be due to the different indicators for measuring the mediating variable of diving attitudes. Notably, the studies by Ong and Musa [12] and Ong and Musa [15] examined the incorporation of divers’ knowledge, dedication, and awareness to assess their attitudes about engaging in scuba diving in Malaysia. Comparatively, the present study focused more on divers’ environmental responsibility awareness as reflected by psychological changes to assess their diving attitude. Therefore, the various indicators used to measure divers’ diving attitudes may have contributed significantly to the different research findings regarding the mediating effects of diving attitude in understanding the relationship between diving experience and responsible underwater behaviour. In addition to the perceived difference in the measurement indicators of diving attitude, cultural differences may contribute significantly to the changes in individuals’ diving attitude, and the changes in individuals’ attitudes caused by cultural differences may have resulted in different research findings between the current study and the previous studies by Ong and Musa [12] and Ong and Musa [15], which were conducted in Malaysia. This assumption is somewhat supported by Johnson, et al. [51], who found that individuals’ environmental beliefs and behaviours could significantly differ from cultural differences, especially regarding their ethnicity, when comparing White Americans to the other four groups of ethnic minorities using national-level data from the United States. This is especially evident when comparing the analysis of respondents’ backgrounds in the current research with those of previous studies conducted in Malaysia. Methodologically formulated, participants in the present study focused primarily on Chinese scuba divers in Hong Kong. In contrast, the research conducted in Malaysia has included both domestic and foreign scuba divers. From a logical standpoint, the inclusion of different racial backgrounds may have significantly impacted divers’ attitudes and behaviour, leading to notable discrepancies between the current and previous investigations in assessing mediation effects.

## 6. Conclusions and Implications

This study examines divers’ responsible underwater behaviour through the measurement variables of their demographic characteristics, diving experience, and attitudes. Alternatively, divers’ attitude was used as a mediating variable to investigate its influence in studying the relationship between diving experience and their responsible underwater behaviour based on the research framework of Ong and Musa [12] and Ong and Musa [15]. According to the research hypothesis, this study has provided valuable insights into understanding divers’ responsible diving behaviour. Theoretically, the relationship between divers’ demographic features, diving attitudes, and responsible underwater behaviour was significantly confirmed. These findings have yet to be found much in Hong Kong, which may help to extend the theoretical framework concerning the development of scuba diving as a particular type of nature-based tourism in Hong Kong. By confirming significant relationships between divers’ responsible underwater behaviour and variations in their demographic features, diving experience, and attitudes, the present study has effectively broadened the spectrum of determinants associated with multi-theoretical variables to understand the determinant factors influencing divers’ responsible diving behaviour while diving in Hong Kong. As expected, the integration of scuba divers’ demographic characteristics, diving experience, and diving attitude is expected to enhance the theoretical understanding of the dynamic factors in shaping scuba divers’ behaviours, which may help to provide a tremendous theoretical foundation for researchers to explore further scholarly work based on the current established theoretical direction in the event of supporting the sustainable marine conservation and tourism development in Hong Kong or even beyond the current geographical location. More importantly, these outcomes may provide scientific evidence for authorities to develop proper management measures for improving divers’ responsible underwater behaviour by acknowledging the differences in divers’ demographic backgrounds, diving experience, and diving attitudes.

As such, several management implications were proposed based on the hypothesis results to help stakeholders manage and maintain the sustainable development of the scuba diving industry, marine ecology, and environmental protection. First, this study concludes that a positive relationship exists between divers’ demographic background and their responsible underwater behaviour, especially with younger and relatively less educated divers who may be more likely to exhibit irresponsible underwater behaviour. Therefore, stakeholders are advised to be closely monitored and intervene proactively with younger and less educated divers to reduce the damage to the marine ecosystem caused by this group of divers. Efforts can focus on providing additional supervision by appointing an experienced diving instructor to accompany and guide those younger and less educated divers to ensure they comply with the recreational regulations and environmental guidelines while diving in marine protected areas. This implication has proven somewhat effective based on the investigation of Hammerton and Bucher [6], who found that open-water divers who were well supervised in water by diving instructors made less contact than more experienced divers, including divemasters. Their findings support the effectiveness of in-water reinforcement applied by experienced scuba instructors to improve divers’ responsible underwater behaviour, thereby reducing damage to marine ecology. In line with the second hypothesis, this study demonstrates a positive relationship between divers’ diving experience and their responsible diving behaviour, indicating that experienced divers may behave more responsibly than less experienced divers while diving in Hong Kong. Thus, authorities are advised to provide predive training programs to divers, especially those with less diving experience. This implementation is based on the findings of Hammerton [52], who found that divers show significantly less exposure to marine life, regardless of their level of diving experience, after participating in a lower-impact diver training program. In the same study, the author also demonstrates that lower-impact diving training techniques could allow divers of all experience levels to learn and apply these techniques to reduce the negative impact of divers on the marine ecosystem. Unsurprisingly, this implication is further supported by the exploration of Giglio, Luiz, Chadwick and Ferreira [7], who claimed that the video briefing proved to be an efficient strategy in mitigating the negative impacts of scuba diving and found that divers at all experience levels reduced their irresponsible diving behaviour after viewing the video, especially those who equipped themselves with cameras while diving. By the end of this study, a positive relationship between divers’ diving attitude and their responsible underwater behaviour was confirmed, indicating that divers with great environmental awareness may behave more responsibly while diving in Hong Kong. Therefore, management authorities and relevant stakeholders are advised to consider offering marine ecology and environmental preservation education programmes to help increase divers’ ecological awareness and knowledge, as previous studies have indicated that environmental awareness education programs may help upgrade recreationists’ environmental attitudes toward conservation [5,13,14]. This would also better assist divers in comprehending the importance of marine ecology and environmental conservation to develop better environmental attitudes to help them avoid irresponsible recreational diving behaviour.

## 7. Limitations and Future Studies

The research findings and the suggested implications of this study have demonstrated profound evidence in helping fulfil the research gaps while contributing to the practical management recommendations associated with sustainable scuba diving tourism development in Hong Kong. However, the present study is relatively simple compared to the variables accommodated in some of the previous studies (structural equation model). This may result in a lack of understanding of other potential factors that influence divers’ responsible underwater behaviour. Specifically, previous studies have used variables such as the diver’s personality [12], environmental concern [15] and recreational specialization [20,21,22] to explore their association with divers’ responsible underwater behaviour across different research sites. Thus, future studies may consider adding more research variables to enhance our understanding of the alternative potential factors influencing divers’ responsible diving behaviour. This will compensate for the current study’s lack of variables in understanding the responsible underwater behaviour variables among scuba divers in Hong Kong.

In addition, the present study mainly used a questionnaire to assess the impact of proposed variables on divers’ responsible diving behaviours, which may not function objectively in response to discovering changes in divers’ diving behaviours. However, previous studies have assessed divers’ responsible underwater behaviour using interviews and underwater observation methodologies, yielding significant study findings across many research sites [5,8,18,23]. As a result, future studies may apply quantitative and qualitative methods to achieve a complementary scientific analytical approach to thoroughly examine the elements associated with impacting divers’ responsible underwater behaviour in Hong Kong.

Finally, the distribution of questionnaires to recreational divers did not reach the level of visitation at the peak volume due to restrictions related to the pandemic movement order. In addition, the questionnaire collection mainly focused on the summer season and excluded divers in other seasons. Therefore, we suggest that future studies occur after the pandemic has subsided, when research data can be collected more systematically to allow for a more extensive study sample.

## Figures and Tables

**Table 1:** Demographic characteristics of the recreational divers.

Variables	Categories	Frequency	Percentage %
Gender	Male	193	48.5
	Female	205	51.5
Age	18–29	182	45.7
	30–39	123	30.9
	40–49	62	15.6
	50–59	27	6.8
	60 or above	4	1.0
Education	Primary	0	0
	Secondary level	77	19.3
	Undergraduate	229	57.5
	Postgraduate	92	23.1
Salary	9999 or below	24	6.0
	10,000–19,999	92	23.1
	20,000–29,999	104	26.1
	30,000–39,999	54	13.6
	40,000–49,999	32	8.0
	50,000–59,999	17	4.3
	60,000 or above	23	5.8
	Retire	26	6.5
	Do not answer	26	6.5
Total	/	398	100

**Table 2:** Exploratory factor analysis.

Constructs	Measurement	Factor Loadings		
		1	2	3
Diving experience	1. When did you acquire your first diving qualification?	0.841		
	2. Number of dives since you acquired qualification.	0.903		
	3. Highest level of diving qualification you have.	0.820		
	4. How many dives in places outside of Hong Kong?	0.867		
Diving attitude	1. I am happy when acting responsibly while diving.		0.776	
	2. I should help to improve diving sites when diving in the MPAs.		0.797	
	3. It is worth making some sacrifices to protect diving sites.		0.778	
Responsible underwater behaviour	1. I will intervene with other divers I encounter harming marine organisms.			0.600
	2. I have been actively helping to clean up the ocean.			0.712
	3. I avoid using chemicals when I am diving (e.g., sunscreen)			0.544
	4. I have helped monitor the beach, seabed, and coral reefs for harmful activity.			0.743
Eigenvalue		3.669	2.203	1.132
% of variance explained		28.02	17.92	17.73
Cronbach alpha		0.90	0.71	0.62

Variance explained = 63.67%, KMO = 0.79, Bartlett’s test of sphericity = *x*^2^(55) = 1686.67; *p* < 0.001, Varimax Rotation Method.

**Table 3:** Confirmatory factor analysis.

Constructs	Indicators Loadings	Factor Loading	AVE	CR.
Diving experiences	1. When did you acquire your first diving qualification?	0.76	0.69	0.90
	2. Number of dives since you acquired qualification.	0.95		
	3. Highest level of diving qualification you have.	0.83		
	4. How many dives in places outside of Hong Kong?	0.77		
Diving attitudes	1. I am happy to act responsibly when diving.	0.51	0.50	0.74
	2. I should help to improve diving sites when diving in the MPAs.	0.76		
	3. It is worth some sacrifices to protect diving sites.	0.81		
Responsible underwater behaviour	1. I will intervene with other divers I see harming marine organisms.	0.45	0.32	0.64
	2. I have been actively helping to clean up the ocean.	0.76		
	3. I avoid using chemicals when I am diving (e.g., sunscreen)	0.46		
	4. I have helped monitor the beach, seabed, and coral reefs for harmful activity.	0.53		

Model fit indices: *χ*^2^ = 130.259, *df* = 41, *x*^2^/*df* = 3.177, *p* < 0.001, *RMSEA* = 0.074, *CFI* = 0.946, *GFI* = 0.945, and *NFI* = 0.924.

**Table 4:** Discriminant validity.

Construct		Mean.	SD.	1.	2.	3.
1.	Diving experience	2.91	1.13	**0.83**		
2.	Diving attitudes	4.65	0.49	0.05	**0.71**	
3.	Responsible underwater behaviour	3.76	0.70	0.49 ***	0.46 ***	**0.56**

*** Correlation is significant at the 0.001 level (2-tailed).

**Table 5:** Heterotrait–monotrait (HTMT) criterion.

Construct	Diving Attitudes	Responsible Behaviour	Diving Experience
Diving attitudes			
Responsible behaviour	0.329		
Diving experience	0.056	0.368	

**Table 6:** Multiple regression between divers’ sociodemographic variables and responsible underwater behaviours.

Variables	Unstandardized		Unstandardized	*t*	Sig.	*R*	*R* ^2^	Δ*R*^2^	*df*	*N*	*F*	*F* Statistics
	Coefficients		Coefficients									
	*B*	*SE*	*Beta* (*β*)									
Gender	0.09	0.07	0.06	1.24	0.22	0.19	0.04	0.03	4	393	3.79	0.005 **
Age	0.10 *	0.04	0.14	2.55	0.01							
Education	0.15 *	0.05	0.14	2.71	0.01							
Salary	−0.03	0.02	−0.10	−1.92	0.06							

Note: *p* < 0.05 *, *p* < 0.01 **.

**Table 7:** Path analysis.

Measurements	*β*	*SE*	*R* ^2^	*t*	*p*
*Regress DV on IV (Path c)*					
Diving experience → Responsible underwater behaviour	0.228	0.029	0.136	7.903	<0.001 ***
*Regress DV on mediator (Path a)*					
Diving experience → Diving attitude	0.024	0.022	0.003	1.085	0.279
*Regress mediator on DV (Path b)*					
Diving attitude → Responsible underwater behaviour	0.429	0.063	0.226	6.776	<0.001 ***

Note: *p* < 0.001 ***.

**Table 8:** Results of the bootstrapping method for mediation.

Effects		Coefficient	Boot SE	95% Biased		Results
				Confidence Interval		
				LLCI	ULCI	
Indirect effects	DE → DA → RUB	0.010	0.009	−0.007	0.028	No mediation
Direct effects	DE → RUB	0.218 ***	0.027	0.164	0.272	/

Note: *** *p* < 0.001. DE = Diving experiences, RUB = Responsible underwater behaviour, DA = Diving attitudes.

## Data Availability

The data presented in this study are available upon request from the corresponding author. The data are not publicly available due to the data also forming part of an ongoing study and cannot be publicly shared for the time being.
